# Hsa_circ_0001492 regulates the hsa-miR-145-5p/ovarian carcinoma immunoreactive antigen domain 2 axis to promote the progression of lung adenocarcinoma

**DOI:** 10.17305/bb.2024.11140

**Published:** 2024-10-26

**Authors:** Yuanqiang He, Gang Li, Ran Fu, Yue Li, Ying Wang

**Affiliations:** 1 Department of Respiratory and Critical Care Medicine, Huai’an Second People’s Hospital, The Affiliated Huai’an Hospital of Xuzhou Medical University, Huai’an City, China

**Keywords:** Circular RNA (circRNA), hsa_circ_0001492, hsa:miR-145-5p, ovarian carcinoma immunoreactive antigen domain 2 (OCIAD2), lung adenocarcinoma (LUAD)

## Abstract

Circular RNA (circRNA) has been proven to be a key regulator in a range of tumor illnesses, such as lung adenocarcinoma (LUAD); however, the regulatory mechanisms of circRNA remain unclear. In this study, circRNA (hsa_circ_0001492) in LUAD was examined for its regulatory and functional potential. Quantitative real-time polymerase chain reaction was used to assess the hsa_circ_0001492 level in LUAD. The RNAse R digestion test was employed to isolate hsa_circ_0001492. The primary location of hsa_circ_0001492 enrichment in LUAD cells was identified through a nucleoplasmic separation test. LUAD cell migration, proliferation, and spherogenicity were examined using wound healing, transwell, EdU, and cell spherogenicity assays. The association between miR-145-5p and hsa_circ_0001492/ovarian carcinoma immunoreactive antigen domain 2 (OCIAD2) was validated using a dual luciferase experiment. The interaction between sh-hsa_circ_0001492 and miR-145-5p was confirmed through an RNA pull-down assay. The effects of hsa_circ_0001492, miR-145-5p, and OCIAD2 on LUAD tumor development were examined using xenograft mouse models and immunohistochemistry tests. Results showed a higher amount of hsa_circ_0001492 in LUAD. The cytoplasm of LUAD cells was observed in the area where hsa_circ_0001492 mainly accumulated; hsa_circ_0001492 enhanced LUAD cell migration, proliferation, and sphere-forming ability. MiR-145-5p and OCIAD2 were identified as targets of hsa_circ_0001492 and miR-145-5p, respectively. The level of OCIAD2 was increased by hsa_circ_0001492 through targeted binding to miR-145-5p. In nude mice, tumor growth was inhibited by silencing hsa_circ_0001492, while knockdown of miR-145-5p and overexpression of OCIAD2 promoted the growth of LUAD tumors. In conclusion, hsa_circ_0001492 regulates the hsa-miR-145-5p/OCIAD2 axis to promote the progression of LUAD, and could be a useful target for the diagnosis and treatment of LUAD.

## Introduction

Lung cancer is a malignant lung tumor with the highest morbidity and mortality rates among malignant tumors in China [1]. Lung adenocarcinoma (LUAD) accounts for 40% of cases and is the most common pathological type of lung cancer [2, 3]. Most LUAD patients are only identified after the tumor has developed locally or has progressed significantly, resulting in a high mortality rate [4, 5]. The prognosis for LUAD has improved with the steady advancement of molecularly targeted medications and therapies. However, due to differences across patient cases, as well as disease severity and resistance to targeted therapy, LUAD prognosis remains unsatisfactory [6–9]. Accordingly, it is necessary to identify potential LUAD pathogenic mechanisms and therapeutic targets to improve treatment outcomes.

Unlike conventional linear RNAs, circular RNAs (circRNAs) are created through back-splicing. They have a unique looped structure that makes them resistant to RNA exonucleases, ensuring a high level of conservation and stability [10, 11]. Numerous studies have shown that circRNAs can act as microRNA (miRNA) sponges, inhibiting miRNA function [12, 13]. CircRNAs are also important regulators of gene expression in many tumors and diseases [14], including LUAD [15], hepatocellular carcinoma [16, 17], breast cancer [18, 19], prostate cancer [20], gastric cancer [21], bladder cancer [22], and glioblastoma [23], among others. These findings suggest that circRNA may play a significant role in the non-coding RNA regulatory network of the cell. Identifying the functional mechanisms of circRNAs is crucial for developing new diagnostic and therapeutic approaches for LUAD.

Through next-generation sequencing, we identified the upregulated circRNA in LUAD as hsa_circ_0001492. Based on database predictions and preliminary experimental results, we hypothesized that hsa_circ_0001492 could target and regulate miR-145-5p, and that miR-145-5p targets the ovarian carcinoma immunoreactive antigen domain 2 (OCIAD2) gene. To further understand the role of hsa_circ_0001492 in LUAD and its molecular regulatory system, we conducted several experiments. Hsa_circ_0001492 expression in LUAD was measured using quantitative real-time polymerase chain reaction (qRT-PCR). Using bioinformatic analysis, dual-luciferase assays, immunohistochemistry (IHC) staining, and western blot analysis, we created hsa_circ_0001492-silenced LUAD cell lines and conducted *in vitro* cellular experiments and *in vivo* nude mouse subcutaneous tumorigenicity experiments to investigate the effects of hsa_circ_0001492 on the growth and function of LUAD cells as well as on tumor development. The findings will contribute to understanding the regulatory network of competing endogenous RNAs in LUAD and provide a theoretical foundation for developing new diagnostic markers and therapeutic targets for LUAD.

## Materials and methods

### Clinical samples

From March 2022 to March 2023, 47 pairs of lung tumor samples were collected at Huai’an Second People’s Hospital (Huai’an City), with the paraneoplastic tissue located roughly 2 cm from the cancerous tissue’s edge. The patients had not received radiation therapy or chemotherapy and had no history of any other type of tumor. The project was approved by the ethics committee of Huai’an Second People’s Hospital (Huai’an City), and all patients signed an informed consent form after being informed of the potential risks. Tissue samples were immediately transported to the lab in liquid nitrogen and stored as backups in a −80 ^∘^C refrigerator or in liquid nitrogen.

### Cell lines and cell culture

The Shanghai Institute of Biochemistry and Cell Biology, Chinese Academy of Sciences, provided human LUAD cell lines (PC9 and H1975) and human normal lung epithelial cells (BEAS-2B). The cells were grown in a cell culture incubator at 37 ^∘^C with 5% carbon dioxide, and their growth shape and density were evaluated under a microscope. Passage was carried out once cell density reached 80%–90%.

### RNA extraction and qRT-PCR

Total RNA was isolated from tissues and cells using the TRIzol Kit (DP424, TIANGEN, Beijing, China). GenePharma (Shanghai, China) created specific primers for the target and internal reference genes. The PrimeScript RT reagent Kit (RR037B, Takara, Osaka, Japan) was used to reverse transcribe the circRNAs and mRNAs to complementary DNAs (cDNAs), and the miRcute Plus miRNA First-Strand cDNA Kit (4992909, TIANGEN, Beijing, China) was used to reverse transcribe the miRNAs to cDNAs. During the qRT-PCR experiment, circRNA and mRNA were identified using SYBR Green Real-Time PCR Master Mix (4309155, Applied Biosystems, DE, USA), and miRNA was detected using the miRcute Plus miRNA qPCR Kit (4992779, TIANGEN, Beijing, China). The internal reference for circ_000149 was 18S rRNA, OCIAD2 was glyceraldehyde 3-phosphate dehydrogenase (GAPDH), and miR-145-5p was U6. The 2^−ΔΔCt^ method was used to analyze the data. The primers were created as follows:

- hsa_circ_0001492: F: 5′-ACCAGCATCCATTGCAAACC-3′; R: 5′-TGGTACCAACCGCACAAACA-3′
- miR-145-5p: F: 5′-CTCACGGTCCAGTTTTCCCA-3′; R: ACCTCAAGAACAGTATTTCCAGG
- OCIAD2: F: 5′-GGTCCGAGTTAGGGGAGAGT-3′; R: AGAAAACACAAAGGGCCGGA
- 18S rRNA: F: 5′-ACATTGGAACCTGCACACCA-3′; R: CAGGCTTTCCAAAGTCTCATTAACA
- U6: F: 5′-AGAGAAGATTAGCATGGCCCCTG-3′; R: 5′-ATCCAGTGCAGGGTCCGAGG-3′
- GAPDH: F: 5′-CCCACTTCTCTCTAAGGAGAAT-3′; R: 5′-TACACGAAAGCAATGCTATCAC-3’.

### RNAse R resistance assay

RNA was extracted from LUAD cells PC9 and H1975, and two samples were taken from each cell line. RNAse R (R7092M, Beyotime Biotechnology, Shanghai, China) was added to one sample of RNA and processed according to the reagent instructions. The RNAs were incubated in a qRT-PCR instrument (25 min at 37 ^∘^C followed by 10 min at 70 ^∘^C). As a negative control, the other RNA sample was dissolved in the same volume of diethyl pyrocarbonate (D5758, Sigma, St Louis, MO, USA) water (0.1%). After reverse transcription using primers, the expression of circRNA (hsa_circ_0001492) and linear RNA (ERBB2IP mRNA) was quantified by qRT-PCR. ERBB2IP primers: F: 5′-TACCAGCATCCATTGCAAAC-3′; R: 5′-ACCAACCGCACAAACAAACT-3′.

### Nucleus and cytoplasm separation

The PARIS Kit (AM1921, Invitrogen, Austin, TX, USA) was used to extract RNA from the nucleus and cytoplasm. Cells were lysed and centrifuged to obtain nuclear and cytoplasmic fractions. A 2× lysis/binding solution with 100% ethanol was then added, and the mixture was filtered and eluted to extract RNA from the nucleus and cytoplasm, which was subsequently quantified via qRT-PCR [24]. U6 and 18S rRNA were used as internal references in the nucleus and cytoplasm, respectively, to calculate the ratio of hsa_circ_0001492 RNA expression in each as follows:
(1)
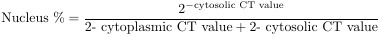


(2)
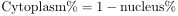
where CT denotes the cycle threshold.

### Cell transfection

The biotin-labeled miRNAs Bio-miR-145-5p and Bio-miR-NC were created by CloudSeq, Inc. (Shanghai, China). The miR-145-5p mimics (miR-145-5p), hsa_circ_0001492 siRNA (si-circ_0001492), miR-145-5p inhibitors (anti-miR-145-5p), OCIAD2 overexpression plasmid (OCIAD2), sh-hsa_circ_0001492-specific lentiviral vectors, and the corresponding negative controls were synthesized and built by GenePharma (Shanghai, China). Following the instruction manual, Lipofectamine 3000 transfection reagent (L3000001, Invitrogen, Austin, TX, USA) was used to transfect the biotin-labeled miR probes and plasmids into PC9 and H1975 cells, respectively, with subsequent experiments performed 48 h later. To create stable transfected LUAD cell lines, well-grown PC9 cells were infected with sh-hsa_circ_0001492-specific lentiviral vectors before being cultured in a complete medium containing 2.0 µg/mL of puromycin (ST551, Beyotime Biotechnology, Shanghai, China) and 40 µL of HiTransG A (REVG004, Genechem, Shanghai, China) infection enhancement solution.

### Wound healing assay

After transfection, phosphate-buffered saline (PBS) was used to resuspend PC9 and H1975 cells, which were then inoculated in six-well plates at a density of 1 × 10^7^ cells per well (1 mL). When the cells flattened out on the bottom, a line was drawn perpendicular to the bottom with a 200-µL pipette tip to create a wound. The cells were then cultured in a serum-free medium for 24 h. Microscopical images were taken at 0 and 24 h. The wound width of cells in the images was calculated using Image J 1.8.0 software (National Institutes of Health, Bethesda, MD, USA) to determine cell mobility in each group. The relative wound width (v. si-NC) of each cell group was then calculated as follows:
(3)
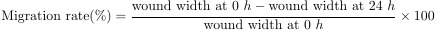


### Transwell assay

After digestion, PC9 and H1975 cells were resuspended in Roswell Park Memorial Institute 1640 medium. Each well of a 24-well plate received 100 µL of the cell suspension, and the lower chamber received 600 µL of complete medium containing 10% fetal bovine serum (C0235, Beyotime Biotechnology). The plate was then kept at 37 ^∘^C for 24 h in a cell culture incubator. The cells were then removed, and the culture medium in the chamber was discarded; the non-migrated cells in the upper inner layer of the chamber were gently wiped with a cotton swab, washed three times with PBS, and fixed with 4% paraformaldehyde (P6148, Sigma) for 30 min before being washed three times with PBS. Cells were stained for 30 min with 0.1% crystal violet staining solution (C0121, Beyotime Biotechnology), rinsed with PBS, dried upside down, and photographed under a microscope at 100× magnification; stained cells were observed and counted.

### EdU assay

An EdU assay (C10310-1, RiboBio, Guangzhou, China) was used to detect cell proliferation [25]; 100 µL of cell suspension was inoculated into 96-well plates (1 × 10^4^ cells/well). Each well received 100 µL of EdU solution (20 µM), and the cells were then incubated for 24 h at 37 ^∘^C. After being fixed with 4% paraformaldehyde, the cells were exposed to the EdU reaction mixture for 30 min at 37 ^∘^C in the dark. They were then washed with PBS, and their nuclei were stained with 4′, 6-diamidino-2-phenylindole (C1005, Beyotime Biotechnology). Fluorescence microscopy was used to record staining results, and EdU incorporation was calculated by randomly observing five high-magnification fields of view.

### Sphere formation assay

The sphere formation assay was conducted as previously described [26, 27]. The transfected PC9 and H1975 cells were resuspended and inoculated into six-well plates containing culture medium, stem cell medium consisting of Dulbecco’s modified Eagle’s medium/F-12 (11320033, Invitrogen), B27 (A3653401, Invitrogen), epidermal growth factor (PHG0313, Gibco), and basic fibroblast growth factor (PHG0368, Gibco), 1 × 10^4^ cells/well (1 mL), without serum. The six-well plates were cultured at 37 ^∘^C and 5% carbon dioxide in a cell culture incubator. After one week of incubation, images were taken under the microscope for inspection; cell spheres were counted, and their sphere formation efficiency was calculated.

### Dual luciferase assay

The anticipated interaction between hsa_circ_0001492/OCIAD2 and miR-145-5p was verified by dual luciferase assay. The wild-type (WT) vector and mutant (MUT) vector were created by inserting the predicted hsa_circ_0001492/OCIAD2 sequence fragment and MUT fragment into the pmirGLO luciferase vector (E1330, Promega, Madison, WI, USA). The two vectors were co-transfected with miR-NC and miR-145-5p into PC9 and H1975 cells, respectively, and cellular luciferase activity was measured 48 h after transfection using the Dual Luciferase Reporter Gene Assay Kit (RG027, Beyotime Biotechnology).

### RNA pull-down assay

The transfected PC9 and H1975 cells (1 × 10^7^) were collected and lysed in lysis buffer (87788, Thermo Fisher, Waltham, MA, USA), and the lysis products were then collected. In the buffer, 3 µL of biotin-labeled miR-145-5p or miR-NC probe was added and incubated at room temperature for 4 h. Thereafter, streptavidin magnetic beads (88817, Thermo Fisher) were added to the buffer and incubated at 4 ^∘^C for 4 h. TRIzol reagent (DP424, TIANGEN) was used to extract the magnetic bead-bound RNA, and qRT-PCR was conducted to determine the hsa_circ_0001492 expression level.

### Western blot

The transfected PC9 and H1975 cells were collected and lysed on ice with a radioimmunoprecipitation assay lysis solution (20101ES60, Yeasen Biotechnology, Shanghai, China) before undergoing centrifugation to extract the proteins. The protein concentration in the supernatant was determined using the Bicinchoninic Acid Protein Kit (P0012, Beyotime Biotechnology). A 10% sodium dodecyl sulfate–polyacrylamide gel was prepared, and protein samples were loaded at 20 µg/well for electrophoresis. The proteins were then transferred to a polyvinylidene difluoride membrane, which was blocked with 5% skimmed milk at room temperature for 1 h after transfer. Rabbit anti-human OCIAD2 (1:1000, ab118565, Abcam, Waltham, MA, USA) and GAPDH (1:1000, ab8245, Abcam) antibodies were added, and the proteins were incubated at 4 ^∘^C overnight. Sheep anti-rabbit immunoglobulin G (1:5000, Abcam, Waltham) was then added after washing with tris-buffered saline + 0.1% Tween 20 (TBST), and the proteins were incubated at room temperature for 30 min. The gray value of each protein band was quantitatively analyzed using Image J 1.8.0 software after washing with TBST and using the ECL Plus Kit (P0018S, Beyotime Biotechnology) under a Chemiluminescence Image Analysis System (5200, Tanon, Shanghai, China).

### Bioinformatics analyses

The hsa_circ_0001492 sequence was queried in the circBase database (http://circrna.org/). Target miRNA prediction for hsa_circ_0001492 was conducted using the circRNA Interactome (CircInteractome; https://circiNTERActome.nia.nih.gov) and CircBank databases (http://www.circbank.cn). For miR-145-5p, the downstream target gene OCIAD2 was predicted using the target gene prediction tools TargetScan (http://www.targetscan.org/), GEPIA (http://gepia.cancer-pku.cn/), and miRPathDB 2.0 (https://mpd.bioinf.uni-sb.de/).

### Xenograft mouse models

Our previously constructed sh-hsa_circ_0001492, sh-NC, sh-hsa_circ_0001492+miR-145-5p inhibitor (i), sh-hsa_circ_0001492+i-NC, sh-hsa_circ_0001492+OCIAD2 overexpression plasmid (OE-OCIAD2), and sh-hsa_circ_0001492 + OE-NC cells (1 × 10^7^ cells/mL) were washed twice with PBS and resuspended in saline, and 12 four- to six-week-old nude mice were prepared (six in each group). The cell suspensions were aspirated with sterile syringes, and 200 µL of the corresponding cell suspension was injected subcutaneously into the right neck of each mouse. Tumor length and width were measured weekly with vernier calipers, and the tumor volume was calculated as follows: (4)
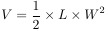
where *L* denotes tumor length and *W* denotes tumor width. The mice were sacrificed five weeks after injection, and the tumors were carefully isolated, weighed, and photographed.

Tumor tissues were fixed in 4% formaldehyde, embedded in paraffin, and cut into 4-µm thick paraffin sections, followed by IHC. Sections were deparaffinized and hydrated with xylene before being incubated for 15 min at room temperature with a 3% hydrogen peroxide solution to inhibit endogenous peroxidase activity. For antigen retrieval, the sections were immersed in citrate antigen retrieval solution (C1032, Solarbio). The sample was blocked with 5% goat serum solution (C0265, Beyotime Biotechnology) for 15 min and subsequently incubated overnight at 4 ^∘^C with mouse anti-human Ki-67 primary antibody (1:1000, ab279653, Abcam, Waltham, MA, USA). The sample was then washed with PBS, followed by the addition of a biotin-labeled sheep anti-mouse immunoglobulin G secondary antibody (1:500, ab150113, Abcam) and incubation at 37 ^∘^C for 1 h before being washed again with PBS. After 10 min of color development in 3,3′-diaminobenzidine solution (P0203, Beyotime Biotechnology), the hematoxylin solution (C0107, Beyotime Biotechnology) was used for counterstaining for 5 min, followed by dehydration in a gradient alcohol solution, clearing with xylene for 5 min, and sealing with neutral resin (G8590, Solarbio). The samples were all stained via IHC alongside a negative control under the same conditions. The staining of tissue sections was observed by microscopy, and Image J 1.8.0 software was used to quantitatively analyze the Ki-67 positive expression rate.

### Ethical statement

The project was approved by the ethics committee of Huai’an Second People’s Hospital (Huai’an City). All patients were required to sign an informed consent form after being informed of the potential risks.

### Statistical analysis

SPSS 26.0 and GraphPad Prism 8.0 software were used for statistical analysis and graphing. Data were expressed as mean ± standard deviation (± s). A normal distribution and homogeneity of variance test were conducted to confirm that the data followed a normal distribution and that the variance was homogeneous. Then, Student’s *t*-test and analysis of variance (ANOVA) were performed to compare different groups, with *P < 0.05 implying statistical significance. Post-hoc comparisons were made using Bonferroni’s method.

## Results

### High expression of hsa_circ_0001492 was detected in LUAD tissues

**Figure 1.**
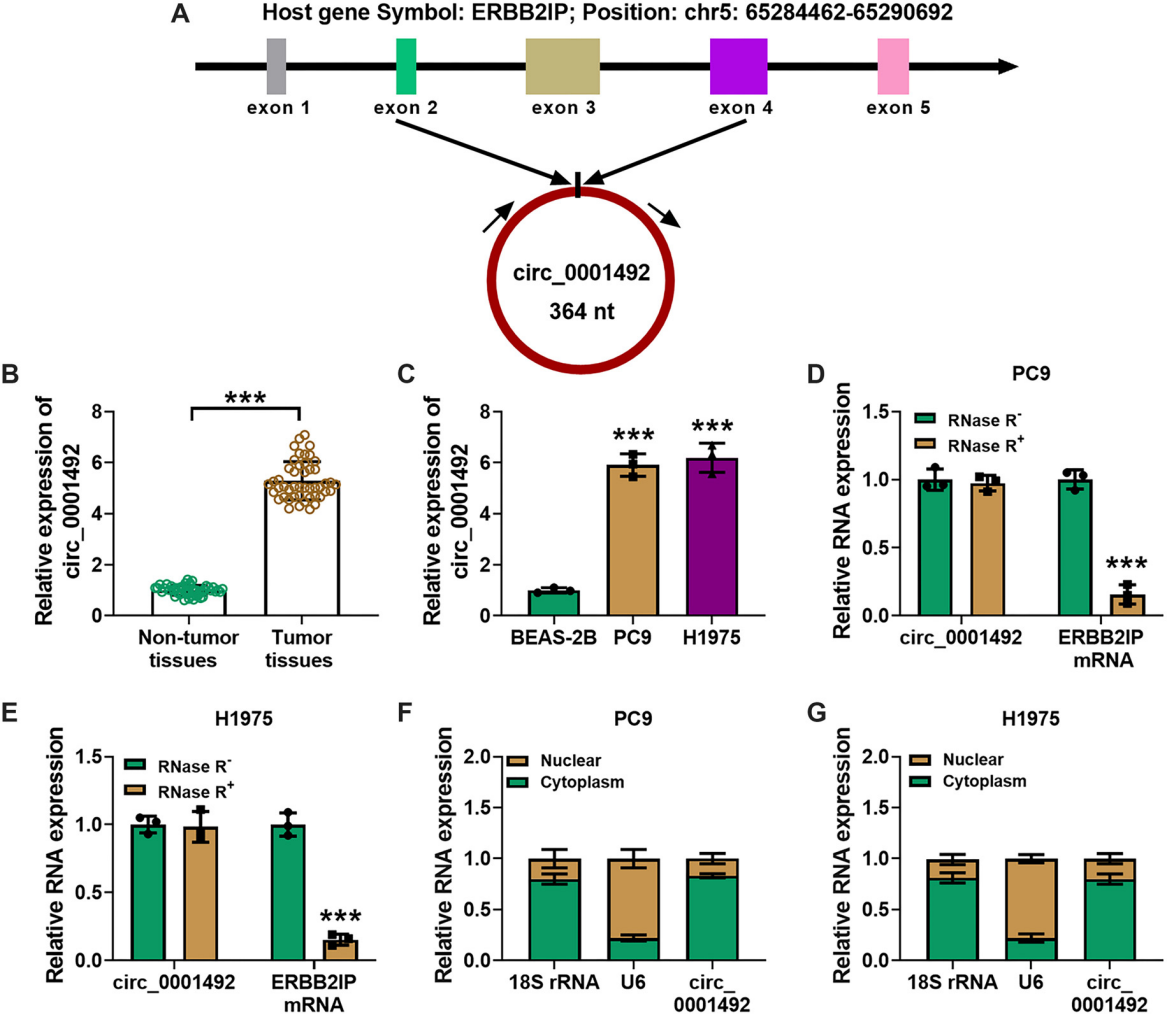
**Hsa_circ_0001492 exhibits a cyclic structure and is abundant in LUAD tissues.** (A) Sanger sequencing confirms the loop structure and gene location of hsa_circ_0001492; (B) Hsa_circ_0001492 expression detected using qRT-PCR in 47 pairs of LUAD and paracancerous tissues; (C) Detection of hsa_circ_0001492 expression levels using qRT-PCR in BEAS-2B and LUAD cell lines (PC9 and H1975); (D and E) Enrichment of hsa_circ_0001492 following RNAse R digestion treatment determined by qRT-PCR; (F and G) Quantification of hsa_circ_0001492 in the nucleus and cytoplasm of LUAD cells determined via qRT-PCR. *n* ═ 3. ***P < 0.001. LUAD: Lung adenocarcinoma; qRT-PCR: Quantitative real-time polymerase chain reaction.

hsa_circ_0001492 was retrieved from the circBase database from chr5: 65284462-65290692 strand: +, with a length of 364 nt, formed by the reverse splicing of exons 2 and 4 of the parental gene ERBB2IP into a loop. Sanger sequencing was performed on the amplification product, and the ring-forming site is depicted in Figure 1A. The results are consistent with circBase. The stability of hsa_circ_0001492 was then confirmed in LUAD tissues and cell lines (PC9 and H1975; Figure 1B and 1C). RNAse R tests showed that hsa_circ_0001492 was significantly resistant to RNAse R (Figure 1D and 1E). Nucleoplasmic separation experiments showed that hsa_circ_0001492 was enriched in the cytoplasm (Figure 1F and 1G).

### The biological characteristics of LUAD cells are hampered by hsa_circ_0001492 silencing

**Figure 2.**
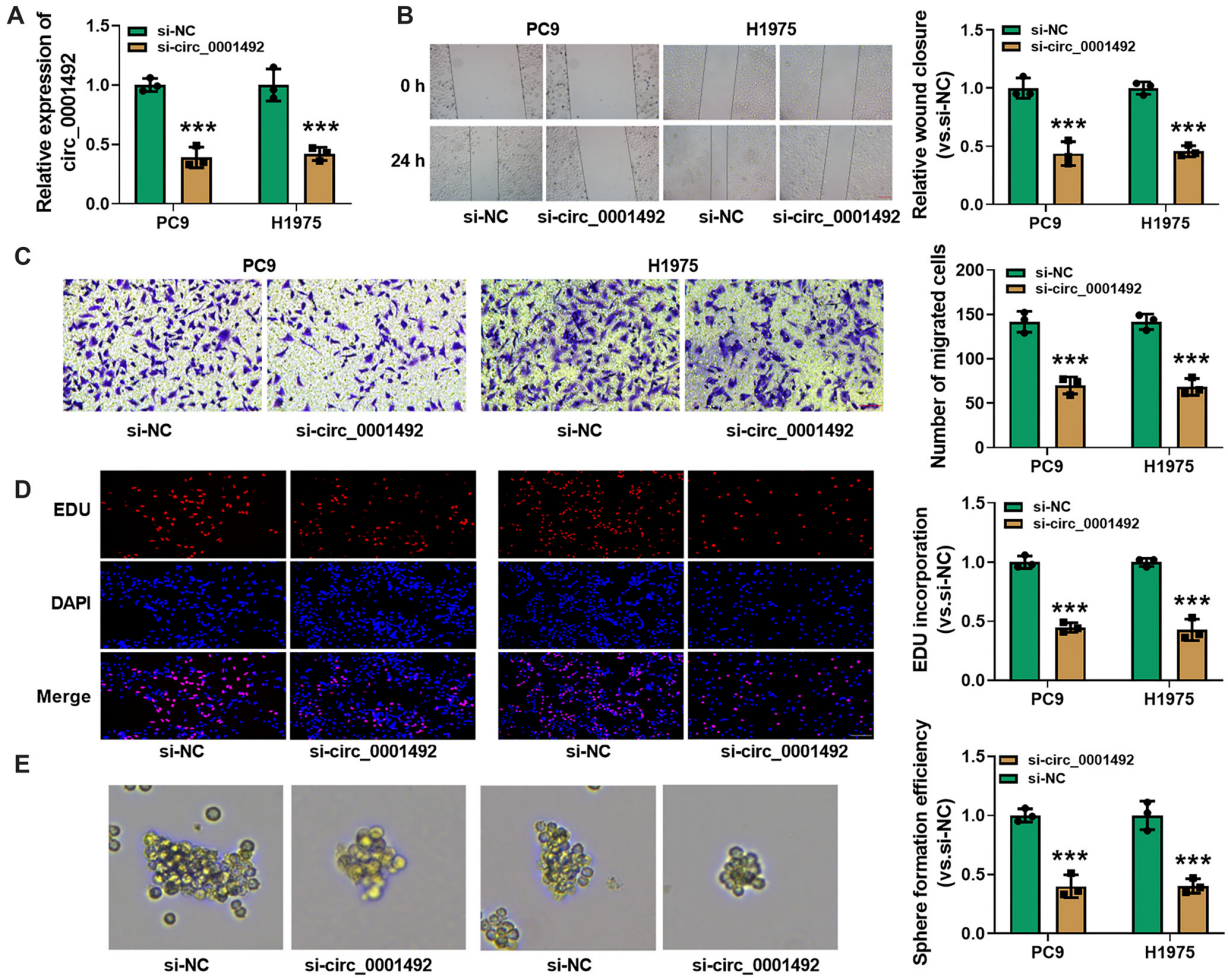
**Silencing hsa_circ_0001492 inhibits LUAD cell migration and proliferation.** (A) Detection of hsa_circ_0001492 expression by qRT-PCR in PC9 and H1975 cells following si-hsa_circ_0001492 transfection; (B) Wound healing test (10×, scale bar ═ 200 µm); (C) Transwell assay (20×, scale bar ═ 100 µm) to assess the migratory potential of PC9 and H1975 cells; (D) EdU assay to examine the effect of si-hsa_circ_0001492 on the proliferation of PC9 and H1975 cells, 20×, scale bar ═ 100 µm; (E) Sphere formation assay to assess sphere formation efficiency in LUAD cells. *n* ═ 3. ***P < 0.001. LUAD: Lung adenocarcinoma; qRT-PCR: Quantitative real-time polymerase chain reaction.

When LUAD cells were transfected with si-hsa_circ_0001492, the qRT-PCR assay revealed that the expression of hsa_circ_0001492 was markedly reduced, as shown in Figure 2A. Wound healing and transwell assays revealed that hsa_circ_0001492 knockdown reduced cell migration (Figure 2B and 2C). The EdU and cell sphere formation assays showed that si-hsa_circ_0001492 transfection inhibited cell proliferation and pelleting ability (Figure 2D and 2E).

### Hsa_circ_0001492 can regulate miR-145-5p expression

**Figure 3.**
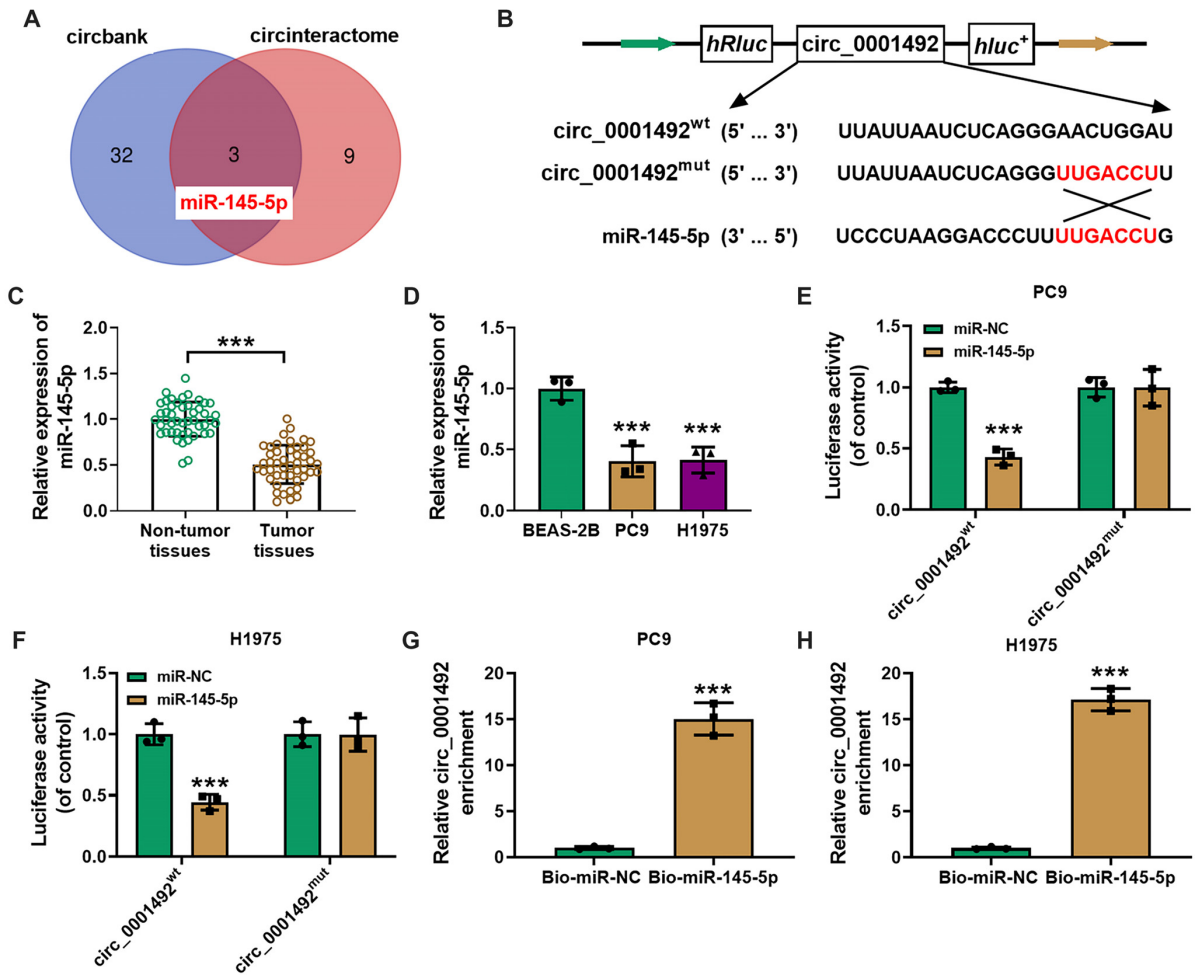
**Molecular prediction and verification of hsa_circ_0001492target miRNAs.** (A) Venn diagram showing miR-145-5p as the predicted target miRNA for hsa_circ_0001492 based on CircInteractome and CircBank; (B) Hsa_circ_0001492 and miR-145-5p binding site; (C) MiR-145-5p expression levels determined using qRT-PCR in 47 pairs of LUAD and paracancerous tissues; (D) MiR-145-5p expression levels determined using qRT-PCR in BEAS-2B and LUAD cell lines (PC9 and H1975); (E and F) Dual luciferase assay to confirm the targeting relationship between hsa_circ_0001492 and miR-145-5p; (G and H) Interaction of hsa_circ_0001492 and miR-145-5p verified via RNA pull-down assay. *n* ═ 3. ***P < 0.001. LUAD: Lung adenocarcinoma; qRT-PCR: Quantitative real-time polymerase chain reaction; miRNA: MicroRNA.

CircRNAs have been shown to act as miRNA sponges, influencing the expression of downstream target genes [28–30]. Based on predictions from the CircInteractome and CircBank databases, three downstream target genes (miR-548c-3p, miR-145-5p, and miR-1263) were identified (Figure 3A). Pre-test data (qRT-PCR of cancer and paracancerous tissues randomly selected from six LUAD patients, which revealed that only miR-145-5p was significantly downregulated in LUAD tissues) and a literature review led to the conclusion that hsa_circ_0001492 can act as a sponge for miR-145-5p. Figure 3B shows the binding location between hsa_circ_0001492 and miR-145-5p. MiR-145-5p expression was downregulated in both LUAD tissues and LUAD cells (PC9 and H1975), in contrast to normal paracancerous tissues and human normal lung epithelial cells (BEAS-2B; Figure 3C and 3D). A dual luciferase assay revealed that, after transfection with hsa_circ_0001492, the relative luciferase activity of WT hsa_circ_0001492^WT^ cells was significantly reduced (*P* < 0.05), whereas MUT hsa_circ_0001492^MUT^ cells did not show significant changes (Figure 3E and 3F). The targeting relationship between hsa_circ_0001492 and miR-145-5p was further supported by RNA pull-down experiments, which revealed that Bio-miR-145-5p enriched hsa_circ_0001492 significantly more than Bio-NC (*P* < 0.05; Figure 3G and 3H).

**Figure 4.**
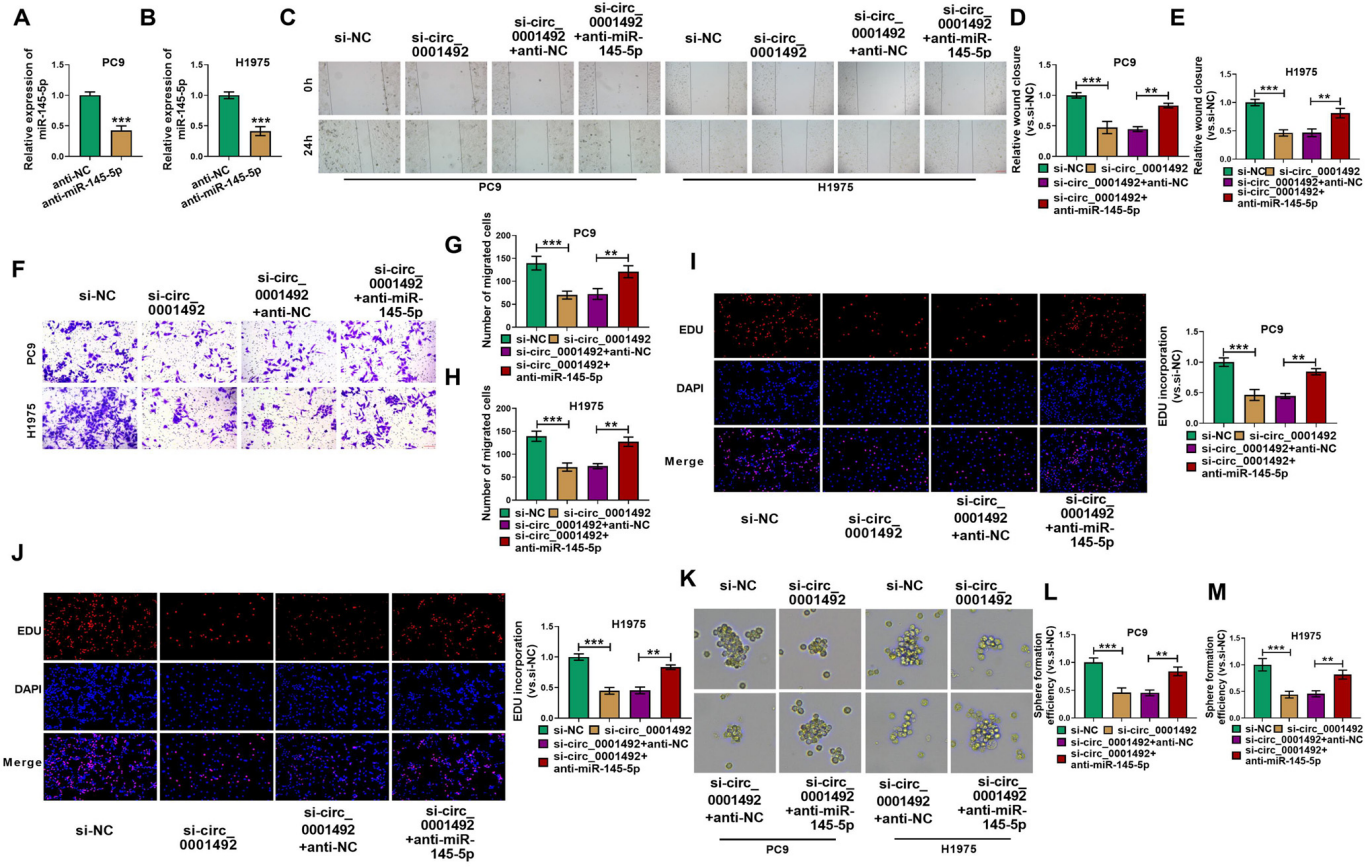
**The effect of hsa_circ_0001492 on LUAD cells is inhibited by miR-145-5p.** (A and B) qRT-PCR assay confirming the inhibitory effect of miR-145-5p on miR-145-5p expression in PC9 and H1975 cells. Wound healing assay; (C–E) and transwell assay; (F–H) showed that the addition of the miR-145-5p inhibitor partially reversed the effect of si-hsa_circ_0001492 on the migratory ability of PC9 and H1975 cells in LUAD; (I and J) EdU assay showing that the miR-145-5p inhibitor partially overrode the inhibitory effect of si-hsa_circ_0001492 on LUAD cell proliferation, 20×, scale bar ═ 100 µm; (K–M) Sphere formation experiment demonstrating that the inhibitory effect of si-hsa_circ_0001492 on the efficiency of sphere formation in LUAD cells is partially reversed by miR-145-5p. *n* ═ 3. **P < 0.01 and ***P < 0.001. LUAD: Lung adenocarcinoma; qRT-PCR: Quantitative real-time polymerase chain reaction.

### MiR-145-5p interferes with the effects of hsa_circ_0001492 silencing on LUAD cells

The expression level of miR-145-5p was significantly downregulated in PC9 and H1975 cells following transfection with the anti-miR-145-5p plasmid, according to a qRT-PCR assay (Figure 4A and 4B). Wound healing and transwell assays demonstrated that si-hsa_circ_0001492 transfection inhibited PC9 and H1975 cell migration; however, when anti-miR-145-5p was concurrently transfected, LUAD cell migration was partially restored (Figure 4C–4H). When anti-miR-145-5p was transfected into LUAD cells, the effects of si-hsa_circ_0001492 on their ability to proliferate and form spheres were partially reversed, as shown in the results of both the EdU assay and the sphere formation assay (Figure 4I–4M).

### MiR-145-5p can regulate OCIAD2 expression

**Figure 5.**
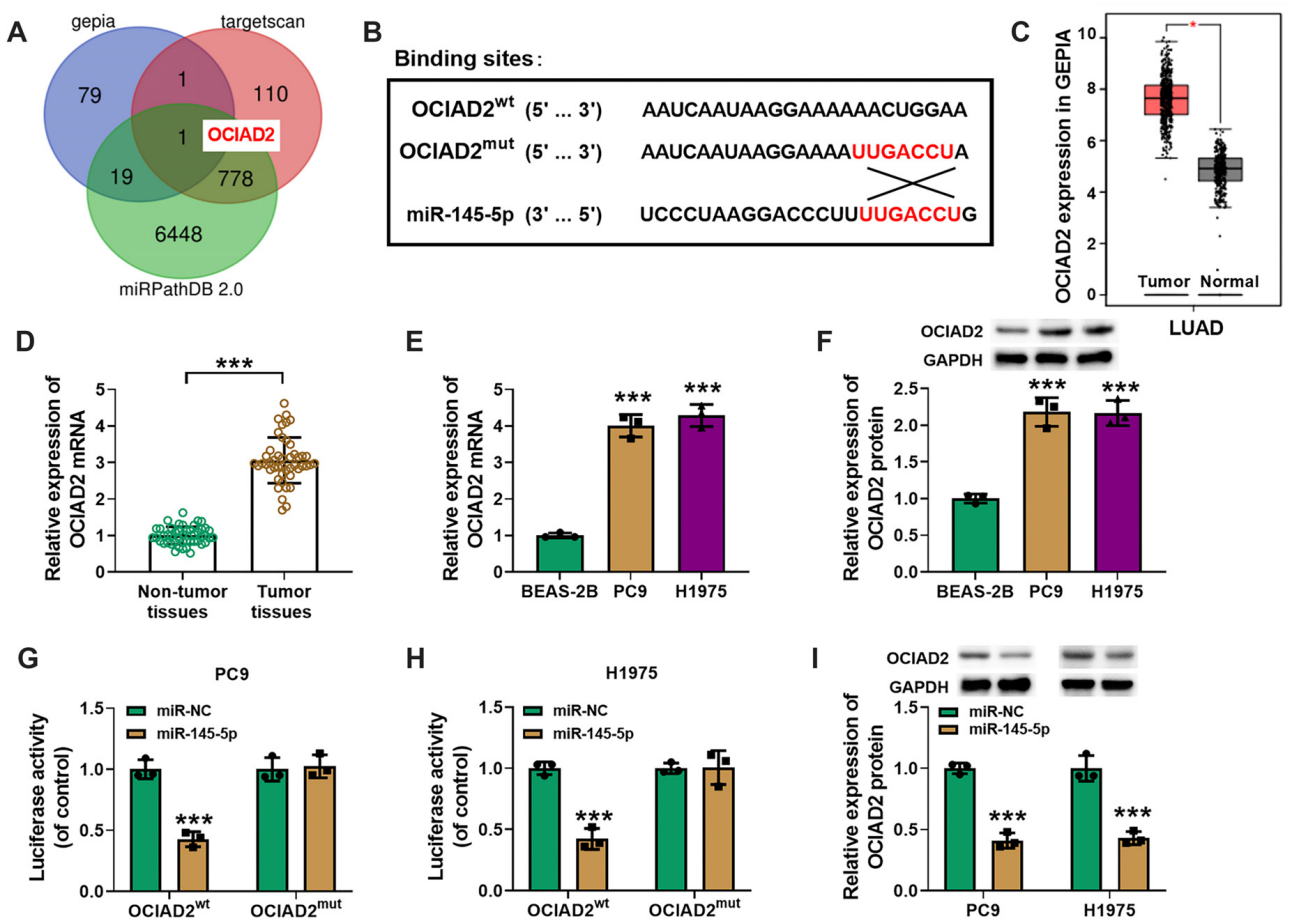
**Molecular prediction and validation of miR-145-5p target mRNA.** (A) The miR-145-5p target identified as OCIAD2 by GEPIA, TargetScan, and the miRPathDB 2.0 databases, depicted in a Venn diagram; (B) OCIAD2 and miR-145-5p binding site; (C) The GEPIA database shows OCIAD2 expression in LUAD and paracancerous tissues; (D) OCIAD2 expression levels in 47 pairs of LUAD and paracancerous tissues measured using qRT-PCR; (E and F) OCIAD2 mRNA and protein expression levels in BEAS-2B and LUAD cell lines (PC9 and H1975) examined using qRT-PCR and western blot analysis; (G and H) Dual luciferase test to validate the association between miR-145-5p and OCIAD2; (I) Western blot analysis of OCIAD2 protein expression in PC9 and H1975 cells treated with miR-145-5p or miR-NC. *n* ═ 3. ***P < 0.001. LUAD: Lung adenocarcinoma; OCIAD2: Ovarian carcinoma immunoreactive antigen domain 2; qRT-PCR: Quantitative real-time polymerase chain reaction.

We continued to predict the target gene of miR-145-5p using the TargetScan, GEPIA, and miRPathDB 2.0 databases to determine potential target genes in LUAD cells; the results suggested that OCIAD2 was the target gene (Figure 5A and 5B). Based on the GEPIA database, LUAD tissues had significantly higher levels of OCIAD2 expression than paracancerous tissues (Figure 5C). In contrast to normal paracancerous tissues and human normal lung epithelial cells (BEAS-2B), OCIAD2 expression was upregulated in both LUAD tissues and cells (PC9 and H1975; Figure 5D–5F). The dual luciferase assay and western blot results showed that miR-145-5p could inhibit OCIAD2 luciferase activity (Figure 5G and 5H) and protein expression in LUAD cells (Figure 5I).

### Hsa_circ_0001492 may enhance OCIAD2 by interacting with miR-145-5p to boost LUAD cell proliferation, migration, and invasion

**Figure 6.**
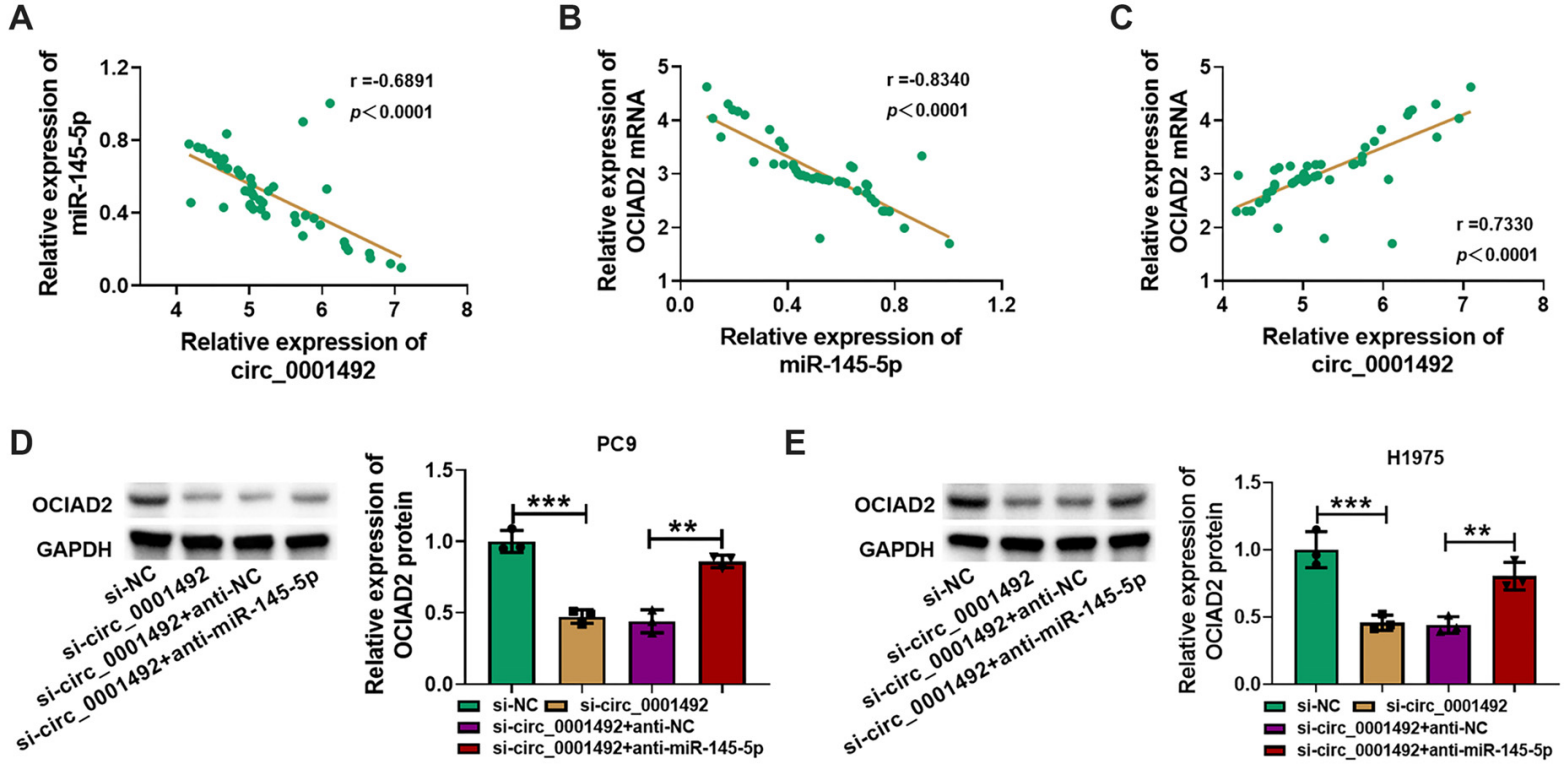
**The relationship and regulation of hsa_circ_0001492, miR-145-5p, and OCIAD2 in LUAD.** (A–C) Pearson correlation analysis of the expression correlation among hsa_circ_0001492, miR-145-5p, and OCIAD2; (D and E) Western blot assay confirms that hsa_circ_0001492 regulates OCIAD2 expression in PC9 and H1975 cells via miR-145-5p. **P < 0.01 and ***P < 0.001. LUAD: Lung adenocarcinoma; OCIAD2: Ovarian carcinoma immunoreactive antigen domain 2.

Pearson correlation analysis suggested that in LUAD tissues, hsa_circ_0001492 expression was negatively correlated with miR-145-5p expression (*r* ═ 0.6891, *P* < 0.0001), miR-145-5p was negatively correlated with OCIAD2 expression (*r* ═ 0.8340, *P* < 0.0001), and hsa_circ_0001492 was positively correlated with OCIAD2 expression (*r* ═ 0.7330, *P* < 0.0001) (Figure 6A–6C). In LUAD cells PC9 and H1975, si-hsa_circ_0001492 alone significantly decreased the protein expression level of OCIAD2 (*P* < 0.05); however, this effect was partially reversed when anti-miR-145-5p was concurrently transfected, leading to upregulation of OCIAD2 protein expression (Figure 6D and 6E).

### Overexpression of OCIAD2 decreases the effect of hsa_circ_0001492 silencing on LUAD cell growth

**Figure 7.**
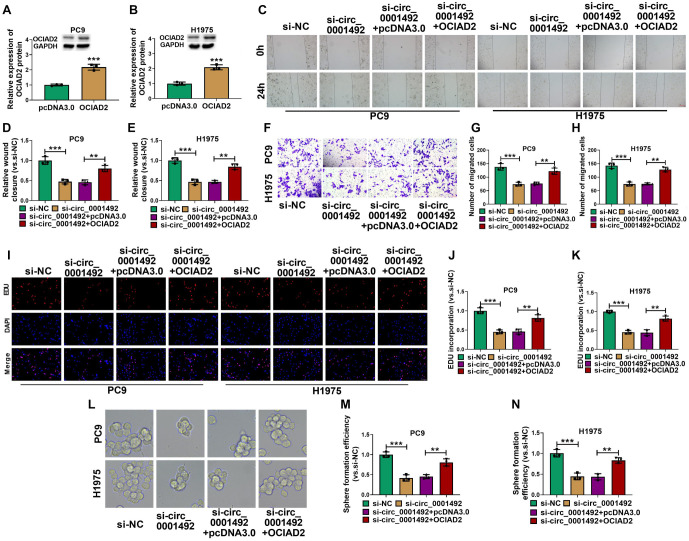
**OCIAD2 overexpression disruptes si-hsa_circ_0001492 regulation of LUAD cells.** (A and B) Western blot analysis showing the effect of the overexpressed OCIAD2 plasmid on OCIAD2 protein expression in PC9 and H1975 cells; (C–E) Wound healing assay; (F–H) Transwell assay showing the effect of OCIAD2 overexpression on the migratory capacity of PC9 and H1975 cells; (I–K) EdU assay examining the effect of OCIAD2 overexpression on PC9 and H1975 cell proliferation; (L–N) Sphere formation test investigating the influence of OCIAD2 overexpression on sphere formation efficiency in PC9 and H1975 cells. *n* ═ 3. **P < 0.01 and ***P < 0.001. LUAD: Lung adenocarcinoma; OCIAD2: Ovarian carcinoma immunoreactive antigen domain 2.

Western blot analysis suggested that the OCIAD2 expression level in LUAD cells significantly increased after transfection with an OCIAD2 overexpression plasmid (Figure 7A and 7B). Transwell and wound healing assays demonstrated that the inhibitory effect of si-hsa_circ_0001492 on LUAD cell migration was partially reversed by OCIAD2 overexpression (Figure 7C–7H). OCIAD2 overexpression similarly reduced the effects of si-hsa_circ_0001492 on the proliferation and spherogenicity of LUAD cells in EdU and sphere formation assays (Figure 7I–7N).

### Knockdown of miR-145-5p or overexpression of OCIAD2 can reverse the inhibitory effect of hsa_circ_0001492 silencing on tumor growth

**Figure 8.**
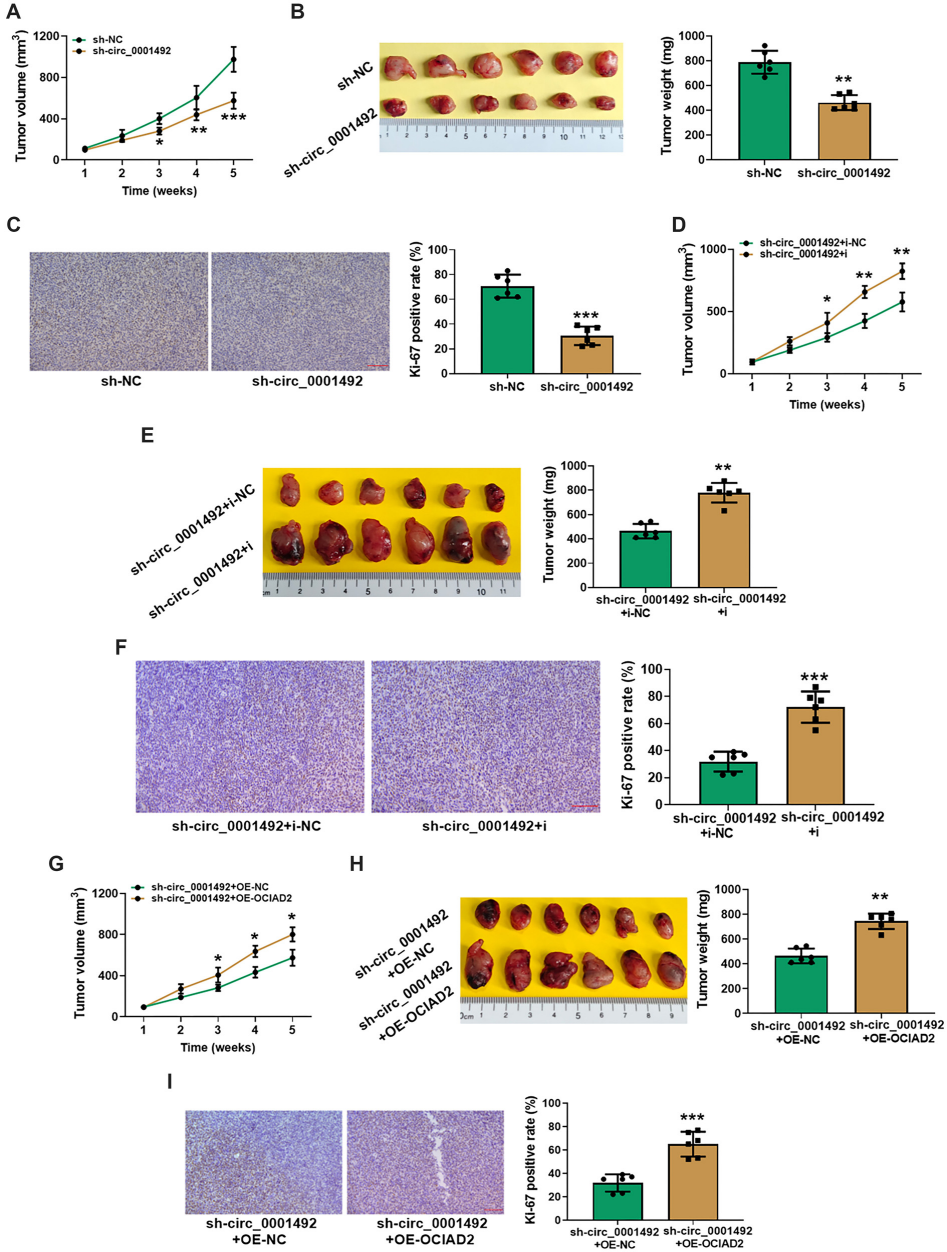
**PC9 cell growth and subcutaneous tumor development in nude mice.** (A, D, and G) Subcutaneous tumor growth rates in nude mice; (B, E, and H) Images of tumors removed from nude mice post-execution (left) and tumor weight (right); (C, F, and I) Ki-67 immunohistochemical micrographs (left) and Ki-67 positivity statistics (right), 20×, scale bar ═ 100 µm. *n* ═ 6. *P < 0.05, **P < 0.01, and ***P < 0.001.

To test the effect of hsa_circ_0001492, miR-145-5p, and OCIAD2 on tumor growth *in vivo*, we injected PC9 cells stably transfected with sh-hsa_circ_0001492, sh-NC, sh-hsa_circ_0001492+i, sh-hsa_circ_0001492+i-NC, sh-hsa_circ_0001492+OE-OCIAD2, or sh-hsa_circ_0001492+OE-NC into the right cervical subcutis of nude mice. After five weeks, the volume and mass of tumors in nude mice in the si-hsa_circ_0001492 group were lower compared to the si-NC group (Figure 8A and 8B). IHC experiments also revealed a significant decrease in Ki-67 positivity in the si-hsa_circ_0001492 group (Figure 8C). Silencing hsa_circ_0001492 expression slowed the growth of LUAD xenograft tumors and inhibited LUAD cell proliferation. Additionally, both knockdown of miR-145-5p and overexpression of OCIAD2 significantly attenuated the inhibitory effect of silencing hsa_circ_0001492 on tumor growth and resulted in significantly higher Ki-67 positivity (Figure 8D–8I). This suggests that both the knockdown of miR-145-5p and the overexpression of OCIAD2 promote the growth of LUAD xenograft tumors and the proliferation of LUAD cells.

## Discussion

As the most common type of lung cancer, LUAD has high incidence and mortality rates. Early symptoms are difficult to diagnose, and effective detection and treatment tools are lacking [2, 31, 32]. The underlying molecular mechanisms of LUAD are yet to be fully clarified; therefore, further research is required concerning LUAD developmental mechanisms at the cellular and molecular levels. This will help with the identification of effective markers to diagnose the early symptoms of LUAD as well as the development of novel therapeutic targets to increase survival rates.

CircRNA is an endogenous non-coding RNA found in eukaryotic cells with a highly stable and conserved closed-loop structure [33]. Studies have suggested that circRNA is a fundamental link between various malignancies, particularly LUAD [34]. CircRNAs primarily serve as miRNA sponges, influencing the miRNA-mediated regulation of gene expression [35, 36], which, in turn, affects tumor cell proliferation, migration, invasion, and drug resistance [37]. For instance, Yang et al. [38] showed that hsa_circ_0007031 (circTUBGCP3) may function as a sponge for miR-885-3p to promote LUAD formation and that hsa-circ-0000211 could upregulate HIF1– expression to enhance LUAD cell migration and invasion [39]. Yu et al. [30] found that hsa_circ_0003258 sponges miR-653-5p, which might accelerate the spread of prostate cancer by upregulating the expression of Rho GTPase-activated protein 5. Similarly, hsa_circ_0004872 has been shown to act as a “molecular sponge” for miR-224, upregulating the expression of miR-224 downstream targets p21 and smad4 and inhibiting the vital activities of gastric cancer cells [40]. Although these studies highlight some of the regulatory mechanisms of circRNAs in cancer, further research into the molecular mechanisms and biological roles of circRNAs in LUAD is required to advance the development of early detection and therapeutic techniques for LUAD malignancies.

After nucleoplasmic separation, we found that hsa_circ_0001492 was present in the cytoplasm of LUAD cells, suggesting that it may function as a competing endogenous RNA to bind miRNAs and control downstream mRNAs. Transfection of si-hsa_circ_0001492 downregulated hsa_circ_0001492 expression in LUAD cells, which, in turn, inhibited cell migration, proliferation, and spheroidogenic ability, according to a qRT-PCR assay. *In vivo* experiments revealed that inhibiting has_circ_0001492 expression delayed tumor growth in nude mice.

Based on database predictions and preliminary experiment results, we hypothesized that miR-145-5p could be a potential binding target for hsa_circ_0001492. MiR-145-5p expression was found to be low in LUAD tissues and cells (PC9 and H1975), implying that miR-145-5p may inhibit the proliferation and metastasis of LUAD tumors. Subsequent dual luciferase and RNA pull-down experiments showed that hsa_circ_0001492 could bind to and inhibit miR-145-5p expression. Pearson correlation analysis demonstrated that miR-145-5p expression was inversely correlated with hsa_circ_0001492, consistent with our prediction. Based on wound healing, transwell, EdU, and cell spheroidization assays, we discovered that anti-miR-145-5p could partially reverse the detrimental effects of si-hsa_circ_0001492 on the migratory, proliferative, and spheroidizing abilities of LUAD cells. These findings suggest that hsa_circ_0001492 can bind miR-145-5p and regulate LUAD development.

Based on database predictions, we found that OCIAD2 might be a binding target for miR-145-5p. OCIAD2 proteins are a conserved class of proteins in eukaryotes, with increased expression in cancerous tumors [41–44]; they are downregulated in hepatocellular carcinoma, gastric carcinoma, glioblastoma, and chronic lymphocytic leukemia [45–49]. OCIAD2 has been associated with tumor formation, though its involvement in LUAD regulation is uncertain. We discovered that miR-145-5p can target and regulate OCIAD2 and that hsa_circ_0001492 can enhance OCIAD2 expression by interacting with miR-145-5p. Pearson correlation analysis suggested that OCIAD2 was positively correlated with hsa_circ_0001492 expression and negatively correlated with miR-145-5p expression. *In vitro* experiments revealed that OCIAD2 overexpression could reduce the effect of si-hsa_circ_0001492 on LUAD cell proliferation while promoting LUAD cell migration, proliferation, and sphere-forming ability. Additionally, *in vivo* experiments revealed that both the knockdown of miR-145-5p and the overexpression of OCIAD2 promoted the growth of LUAD xenograft tumors and the proliferation of LUAD cells.

The results showed that the hsa_circ_0001492 expression level was significantly elevated in LUAD tissues and cells, implying that hsa_circ_0001492 may promote LUAD progression. Both *in vitro* and *in vivo* research demonstrated that inhibiting hsa_circ_0001492 expression prevented the emergence of LUAD. Hsa_circ_0001492 can act as a miR-145-5p sponge, enhancing OCIAD2 expression. The hsa_circ_0001492/miR-145-5p/OCIAD2 axis is thought to provide a new LUAD diagnostic target. Future studies should examine the regulatory role and mechanism of hsa_circ_0001492 in associated pathway proteins. There are still some shortcomings in this research, and in future studies, we can further explore the crosstalk of the hsa_circ_0001492/miR-145-5p/OCIAD2 axis with other signaling pathways.

## Conclusion

This study demonstrated that hsa_circ_0001492 can upregulate OCIAD2 expression to promote LUAD development by acting as a miR-145-5p sponge. This contributes to our understanding of the ceRNA regulatory network in LUAD and establishes a new theoretical foundation for researching novel diagnostic markers and treatment targets for LUAD. Designing new drugs that target the inhibition of hsa_circ_0001492 may provide an effective therapeutic pathway to block tumor metastasis and improve patient prognosis. There are still some shortcomings in this research, such as small sample sizes or potential off-target effects of RNA interference, and the signaling through which hsa_circ_0001492 regulates tumor progression can be further explored in future studies.

## Supplemental data

**Highlights:**

1. Lung adenocarcinoma (LUAD) tissue samples exhibited significantly higher levels of hsa_circ_0001492 expression.
2. MiR-145-5p expression was reduced in LUAD tissue samples.
3. Ovarian carcinoma immunoreactive antigen domain 2 (OCIAD2) expression in LUAD tissue samples was abnormally high.
4. Hsa-miR-145-5p was regulated by hsa_circ_0001492.
5. OCIAD2 was targeted and regulated by hsa-miR-145-5p.
6. Hsa_circ_0001492 regulated OCIAD2 expression through miR-145-5p.

**Graphical abstract.**
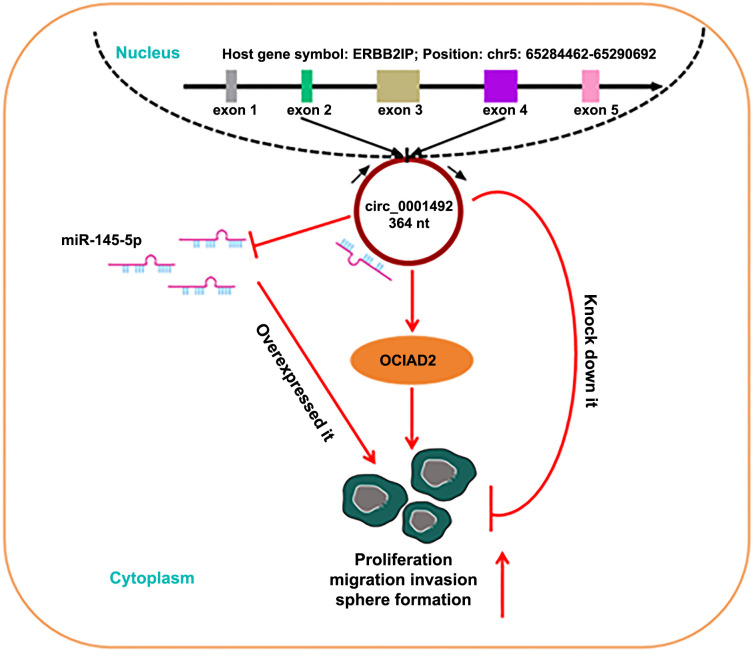
Hsa_circ_0001492 maintains the oncogenic function of OCIAD2 by regulating miR-145-5p, promoting cell proliferation, migration, invasion, and sphere formation, and exacerbating LUAD development.

## Data Availability

The data supporting the findings of this study can be obtained from the corresponding author, upon request.
